# Postharvest Treatment of Hydrogen Sulfide Delays the Softening of Chilean Strawberry Fruit by Downregulating the Expression of Key Genes Involved in Pectin Catabolism

**DOI:** 10.3390/ijms221810008

**Published:** 2021-09-16

**Authors:** Sebastian A. Molinett, Juan F. Alfaro, Felipe A. Sáez, Sebastian Elgueta, María A. Moya-León, Carlos R. Figueroa

**Affiliations:** 1 Laboratory of Bionanotecnology, Instituto de Investigaciones Agropecuarias, INIA CRI La Cruz, Chorrillos 86, La Cruz 2280454, Chile; felipealfaro88@gmail.com; 2 Faculty of Forest Sciences, Universidad de Concepcion, Casilla 160-C, Concepcion 4070386, Chile; felipe.saez.quintana@gmail.com; 3 Núcleo de Investigaciones Aplicadas en Ciencias Veterinarias y Agronómicas, Universidad de Las Américas, Sede Providencia, Santiago 7500975, Chile; selgueta@udla.cl; 4 Institute of Biological Sciences, Campus Talca, Universidad de Talca, Talca 3465548, Chile; alemoya@utalca.cl

**Keywords:** *Fragaria chiloensis*, strawberry decay, shelf-life, cell wall disassembly, pectinases, gasotransmitter

## Abstract

Hydrogen sulfide (H_2_S) plays several physiological roles in plants. Despite the evidence, the role of H_2_S on cell wall disassembly and its implications on fleshy fruit firmness remains unknown. In this work, the effect of H_2_S treatment on the shelf-life, cell wall polymers and cell wall modifying-related gene expression of Chilean strawberry (*Fragaria chiloensis*) fruit was tested during postharvest storage. The treatment with H_2_S prolonged the shelf-life of fruit by an effect of optimal dose. Fruit treated with 0.2 mM H_2_S maintained significantly higher fruit firmness than non-treated fruit, reducing its decay and tripling its shelf-life. Additionally, H_2_S treatment delays pectin degradation throughout the storage period and significantly downregulated the expression of genes encoding for pectinases, such as polygalacturonase, pectate lyase, and expansin. This evidence suggests that H_2_S as a gasotransmitter prolongs the post-harvest shelf-life of the fruit and prevents its fast softening rate by a downregulation of the expression of key pectinase genes, which leads to a decreased pectin degradation.

## 1. Introduction

The Chilean strawberry [*Fragaria chiloensis* (L.) Mill.] is a native species from South America and the maternal progenitor of the commercial strawberry *Fragaria* × *ananassa* Duch. [1]. The Chilean strawberry is a non-climacteric fruit that possesses remarkable organoleptic properties, such as good taste, aroma, nutritional value and an exotic white fruit appearance, having a great potential to become a new exotic berry fruit for the worldwide market [2,3,4]. Besides, *F. chiloensis* fruit is emerging as a new model for studying several ripening-associated processes in strawberries [5,6], such as anthocyanin biosynthesis and plant cell wall disassembly [7,8]. Studies on fruit softening are important as this influences the postharvest life of highly perishable fruit [9].

The ripening-associated softening of fleshy fruit has been largely described as a direct consequence of enzyme-mediated cell wall disassembly [10]. Events such as depolymerization and solubilization of hemicelluloses and pectins within the cell wall often occur in many fleshy fruits, including strawberry [7]. Genes encoding for proteins involved in cell wall disassembly, such as polygalacturonase (PG), pectate lyase (PL), pectin methylesterase (PE), endo-β-1,4-glucanase (EG), β-galactosidase and expansins (EXPs), increase their expression during strawberry ripening [7,11,12,13,14]. Previous reports have shown that Chilean strawberry fruit has a faster-softening rate than *F.* × *ananassa* ‘Chandler’ fruit, which is the major disadvantage for the commercialization of this fruit [12]. The higher softening rate of *F. chiloensis* fruit has been associated with a high expression level of the *PG* gene during fruit ripening [12]. Several reports indicate that pectin metabolism has a significant role in strawberry fruit firmness [7,13,15,16] rather than hemicellulose or cellulose metabolism [17]. However, hemicellulose modification might play a role in Chilean strawberry fruit softening [18]. Regarding the evidence, it is relevant to gain insights about signal molecules that regulate at the biochemical and molecular level and this biological process during the ripening and postharvest of strawberry fruit.

Hydrogen sulfide (H_2_S) is a gas molecule traditionally associated with phytotoxins [19]. However, evidence in animal systems has demonstrated its roles as an endogenous signal molecule and it has been proposed as a gaseous regulator of various physiological functions, similar to nitric oxide (NO) and carbon monoxide (CO) [20,21,22]. Increasing evidence in plants suggests that H_2_S plays several physiological roles, such as enhancer of photosynthesis, stomatal movement, seed germination, root organogenesis, delay senescence of cut flowers and fresh fruit, and biotic and abiotic stress tolerance alone or through interaction with plant hormones [22,23,24,25,26,27,28,29]. Furthermore, H_2_S is biosynthesized in plants from sulfite by sulfite reductase, and from cysteine by L/D-cysteine desulfhydrase and β-cyanoalanine synthase [30,31,32]. Additionally, H_2_S can be removed enzymatically from plant tissue by the action of O-acetylserine (thiol) lyase [33]. Altogether, H_2_S physiological roles and metabolism evidenced in plants suggest that it might have a role as an endogenous gaseous regulator [32]. 

H_2_S-fumigated fruit, using several donors of this gasotransmitter, prolongs its postharvest shelf-life and increases the antioxidant capacity of the tissues, reducing the levels of reactive oxygen species (ROS) and ROS-induced damage [29]. Interestingly, it has also been described as the impact of H_2_S in postharvest physiology of several climacteric (e.g., banana and tomato) and non-climacteric (e.g., grape and strawberry) fruits [34,35,36,37,38]. In banana, H_2_S treatment sustained fruit chlorophyll content, increased carotenoids, soluble proteins, and the overall antioxidant capacity [34]. Authors suggest that H_2_S delayed banana fruit ripening and senescence via an antagonizing effect with ethylene, through the alleviation of oxidative stress and inhibition of ethylene signaling [34]. In this sense, H_2_S application to tomato fruits delayed the acquisition of color and maintained higher chlorophyll and nutritional-related metabolites content during ripening [35]. In the case of non-climacteric fruits, fumigation with H_2_S of grape berries, prior to postharvest storage, preserved in high levels several quality markers such as firmness, soluble solids, titratable acidity (TA), and relevant metabolites such as ascorbic acid, carotenoids, flavonoids, total phenolics, reducing sugars, and soluble proteins [36]. In the same work, H_2_S fumigation reduced the accumulation of ROS and malondialdehyde (MDA) in grape pulp, while it increased the activity of antioxidant enzymes [36]. In strawberry (*F.* × *ananassa*) fruit, postharvest H_2_S treatment also increased the activity of antioxidant-related enzymes and maintained fruit firmness mainly by a decrease of the activity of cell wall-modifying enzymes such as PG, PE, and EG prolonging the shelf-life of strawberries [37,38].

Thus, according to recent evidence, we hypothesized that H_2_S applied at harvest can delay the softening of Chilean strawberry fruit during its postharvest period by affecting cell wall disassembly. In the present work, we study the effect of H_2_S treatment on the metabolism of cell wall polysaccharides, i.e., pectin and hemicellulose catabolism and the implications on the expression of genes encoding for enzymes involved in cell wall modification during postharvest-associated fruit softening.

## 2. Results

### 2.1. Effects of H_2_S Treatment on Postharvest Shelf-Life of Chilean Strawberry Fruit

**Figure 1:**
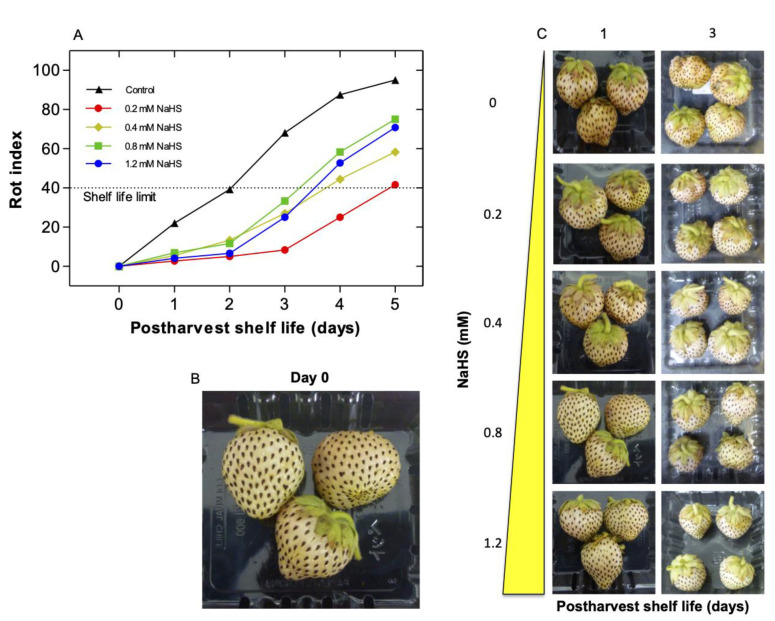
Effects of different H_2_S donor concentrations on rot index and appearance during postharvest shelf-life of Chilean strawberry (*Fragaria chiloensis*) fruit. Strawberries were treated after harvest with H_2_S donor NaHS at different concentrations (0, 0.2, 0.4, 0.8 and 1.2 mM). Changes in (**A**) rot index; (**B**) Appearance of strawberry fruit at harvest and (**C**) after exposure to H_2_S donor NaHS during 3 d of storage at 20 °C.

The effect of H_2_S gas, produced by its donor NaHS, was studied during the shelf-life of Chilean strawberry fruit at 20 °C. Different NaHS concentrations (0.2, 0.4, 0.8 and 1.2 mM) were assayed. Fruit decay was evaluated during the postharvest period and the limit of shelf-life was set at a decay index of 40 (Figure 1A). H_2_S treatment reduces fruit decay and prolongs the postharvest shelf-life of the Chilean strawberry fruit by an effect of optimal dose (Figure 1). Untreated strawberries rapidly decay during shelf-life, reaching the disposal limit after only two days at 20 °C. By contrast, strawberries treated at harvest with NaHS are less prompted to fungal infection and displayed a slower decay.

**Figure 2:**
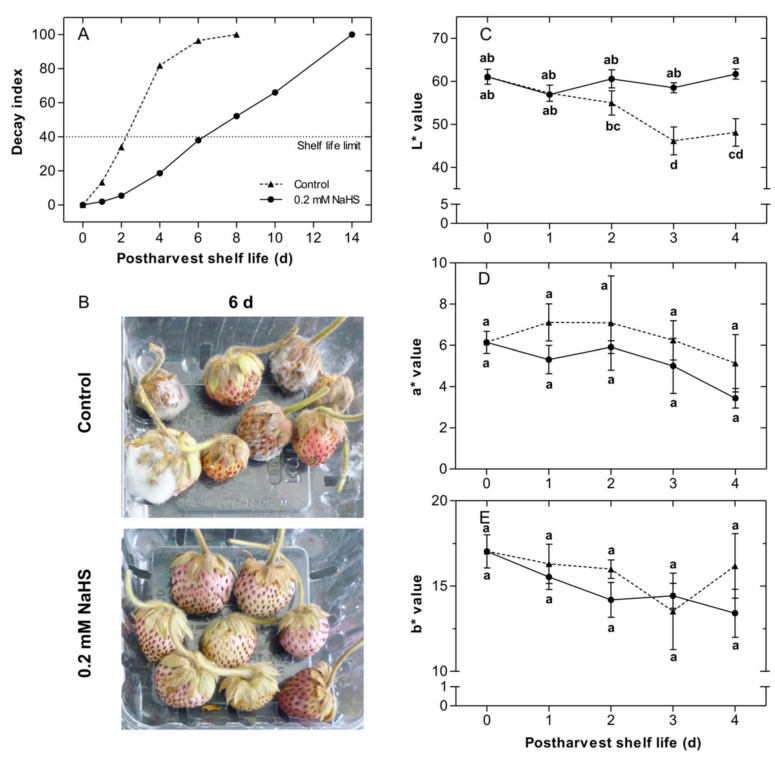
Effects of H_2_S treatment on decay index and color parameters during postharvest shelf-life of Chilean strawberry (*Fragaria chiloensis*) fruit. Strawberries were treated after harvest with 0.2 mM H_2_S donor NaHS. Changes in (**A**) decay index; (**B**) Images of control and H_2_S-treated strawberries after 6 d of storage at 20 °C; (**C**–**E**) Color parameters showing changes in L, a*, b* values, respectively. Each value is the mean of three replicates and vertical bars represent standard errors. Different letters indicate significant differences at *p* < 0.05. For details, see Section 4.

Fruit exposed to 0.2–1.2 mM NaHS solutions displayed a longer shelf-life than untreated fruit, nevertheless, the maximum effect was reached with 0.2 mM NaHS (Figure 1A). In subsequent experiments, 0.2 mM NaHS was employed, extending the shelf-life period (14 days) (Figure 2). NaHS-treated fruit reached their shelf-life limit after 6 d of storage; while untreated fruit reached this stage after 2 d, thus the shelf-life period of NaHS-treated fruit was nearly tripled (Figure 2A). Interestingly as shown in Figure 2B, after 6 d of storage, untreated fruit evidenced severe signs of fungal infection, while NaHS-treated strawberries were not affected.

### 2.2. Effects of H_2_S Treatment on Fruit Color during the Shelf-Life of Chilean Strawberry Fruit

Changes in the external color of strawberries during storage, both in control and NaHS-treated fruit, were evaluated throughout the shelf-life period (Figure 2C). Untreated fruit showed a significant decrease in lightness (L*), while fruit exposed to 0.2 mM NaHS maintained this color parameter during 4 d of shelf-life (Figure 2C). In addition, control and NaHS-treated fruit displayed a similar decreasing pattern of other color parameters (a* and b* values) during shelf-life (Figure 2D,E). Color measurements were not reliable at longer shelf-life periods due to the presence of fungal development on the surface of control strawberries.

### 2.3. Effects of H_2_S Treatment on Fruit Softening and Respiration Rate of Chilean Strawberry Fruit during the Shelf-Life Period

**Figure 3:**
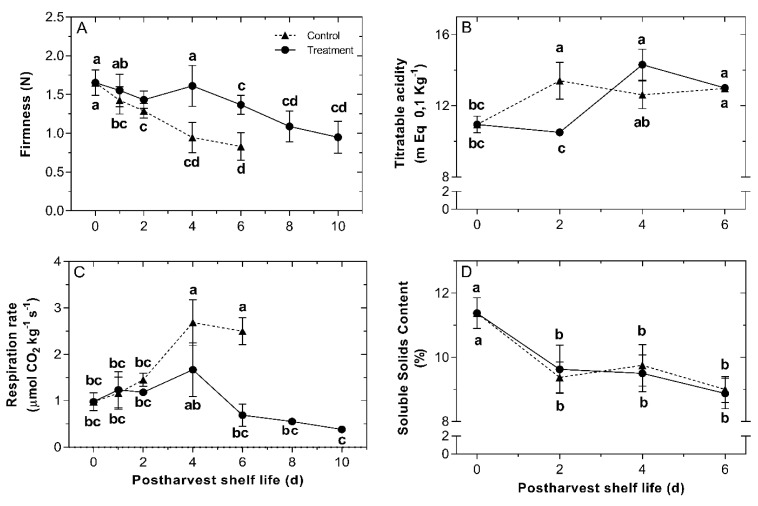
Effects of H_2_S treatment on firmness, titratable acidity, respiration rate and soluble solids content during postharvest shelf-life of Chilean strawberry (*Fragaria chiloensis*) fruit. Strawberries were treated after harvest with 0.2 mM H_2_S donor NaHS. Changes in (**A**) fruit firmness; (**B**) titratable acidity; (**C**) respiration rate, and (**D**) soluble solids content. Each value is the mean of three replicates and vertical bars represent standard errors. Different letters indicate significant differences at *p* < 0.05. For details, see Section 4.

Changes in fruit firmness were followed during the storage period in control and NaHS-treated fruit. Untreated Chilean strawberry fruit evidenced a rapid decrease in firmness throughout the shelf-life period; nevertheless, fruit treated with 0.2 mM NaHS remained firmer than untreated fruit (Figure 3A). Major differences in firmness between treatments were recorded after 4 d at 20 °C, with values of 1.61 N for NaHS-treated fruit and 0.76 N for untreated fruit. Firmness values of untreated fruit were not reliable after 6 d of storage.

As H_2_S is an acid molecule, titratable acidity (TA) was determined to investigate whether exposure to NaHS treatment influences changes in acidity (Figure 3B). TA values increased both in NaHS-treated and control fruit, although with a different pattern. In untreated fruit, TA increases immediately after harvest, while in NaHS-treated fruit, there is a delay as no changes in acidity take place during the first two days. At 6 d of storage, the same acidity level was recorded in both fruit conditions.

Changes in respiration rate were also determined during the shelf-life period (Figure 3C). The production of CO_2_ of NaHS-treated fruit was maintained at a lower rate during shelf-life, and in contrast, it increased constantly in untreated fruit until 4 d. The respiration rate of untreated fruit was not measured at 6 d of the storage period due to the presence of fungus on the fruit surface, which also releases CO_2_, interfering with the determination of the fruit tissue.

Changes in soluble solids content (SSC) were also followed in control and NaHS-treated fruit during shelf-life (Figure 3D). SSC decreased in both fruit groups during shelf-life with no differences.

### 2.4. Effects of H_2_S Treatment on Cell Wall Polymer Solubilization during the Shelf-Life of Chilean Strawberry Fruit

**Figure 4:**
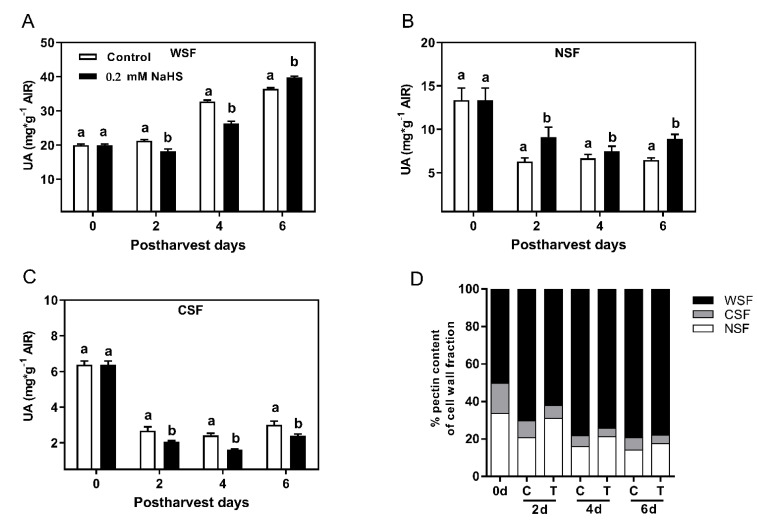
Effects of H_2_S treatment on the content of pectin-related polymers during postharvest shelf-life of Chilean strawberry (*Fragaria chiloensis*) fruit. Strawberries were treated after harvest with 0.2 mM H_2_S donor NaHS, and sampling was performed at harvest (0 d) and after 2, 4 and 6 d of storage at 20 °C. The content of uronic acids (UA) was determined in the water (WSF)-, CDTA (CSF)- and Na_2_CO_3_ (NSF)-soluble cell wall fractions. Changes in (**A**) water-soluble pectins; (**B**) CDTA-soluble pectins; (**C**) Na_2_CO_3_-soluble pectins, and (**D**) the relative content (%) of several pectin fractions in control and H_2_S-treated fruits during postharvest. Values correspond to the mean of three independent cell wall extractions per sampling date. Each value is the mean of three replicates and vertical bars represent standard errors. Different lowercase letters indicate significant differences between control and treatment conditions in each day at *p* < 0.05. For details, see Section 4.

Cell wall fractionation was performed from AIR samples prepared from *F. chiloensis* fruit samples subjected to H_2_S treatment. Total cell wall yield was subjected to a sequential fractionation procedure to separate several pectin fractions. The fractionation of pectins in WSF, CSF, and NSF fractions corresponds to loosely-, ionically-, and covalently-bound pectins, respectively, while KSF is mainly associated with the hemicellulose fraction. During the postharvest period, significantly higher solubilization of pectins was observed in the control fruit than in H_2_S-treated fruit (Table S1, Figure 4). The solubilization of pectins advised as the increment in WSF is accompanied by a decrease in CSF and NSF fractions in non-treated fruit (Figure 4B–D). In H_2_S-treated fruit, pectin solubilization was delayed as WSF did not increase and CSF and NSF did not decrease as in the control fruit. All this evidence suggests that H_2_S treatment delays pectin degradation in Chilean strawberry fruit during the shelf-life period.

Regarding the solubilization of hemicelluloses, an increase in its content was observed in cell wall material obtained from control fruit during the shelf-life period; however, in H_2_S-treated fruit, this increase is delayed in time (Table S1). The content of hemicelluloses of control fruit after 2 d of shelf-life was reached after 6 d of storage by H_2_S-treated fruit.

### 2.5. Effects of H_2_S Treatment on the Expression of Genes Involved in Pectin Degradation during Strawberry Shelf-Life

**Figure 5:**
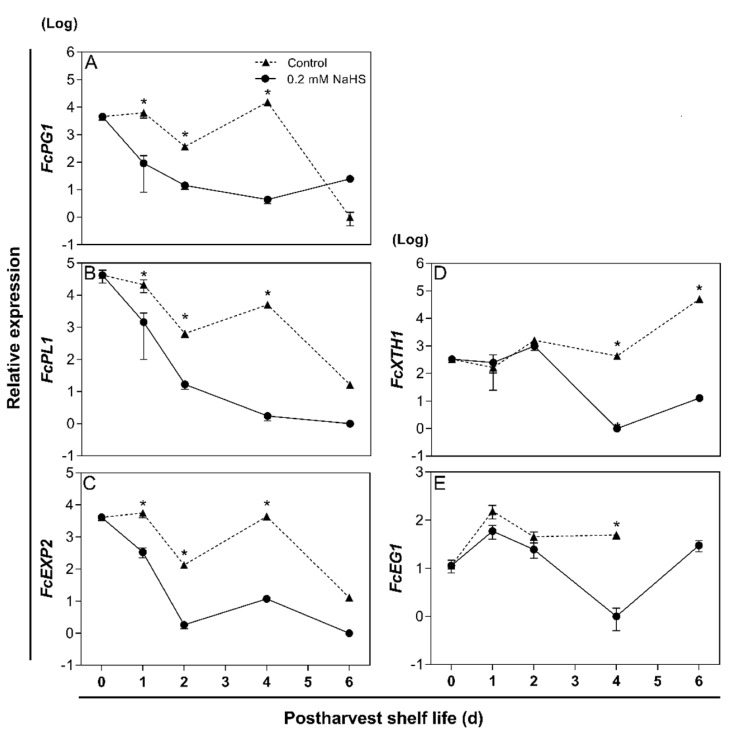
Effects of H_2_S treatment on the expression of cell wall disassembly- and remodeling-related key genes during postharvest shelf-life of Chilean strawberry (*Fragaria chiloensis*) fruit. Strawberries were treated after harvest with 0.2 mM H_2_S donor NaHS and maintained under storage at 20 °C for up to 6 d. Sampling was performed at harvest (0 d) and after 2, 4 and 6 days. Gene expression profile of (**A**) polygalacturonase 1 (*FcPG1*); (**B**) pectate lyase 1 (*FcPL1*); (**C**) expansin 2 (*FcEXP2*); (**D**) xyloglucan transglycosylase-hydrolase 1 (*FcXTH1*), and (**E**) endo-β-1,4-glucanase 1 (*FcEG1*). The data were analyzed by Tukey test (*p* < 0.05) per time. Each value represents mean ± SD (*n* = 4). Asterisks indicate significant differences between treatments. For details, see Section 4.

To gain molecular insights to explain fruit firmness changes and pectin solubilization in H_2_S-treated fruit, the expression of genes involved in cell wall metabolism was analyzed during shelf-life (Figure 5). The expression of genes encoding enzymes involved in pectin solubilization such as polygalacturonase (*FcPG1*) and pectate lyase (*FcPL1*), displayed a drastic and fast reduction in response to H_2_S treatment (Figure 5A,B). By contrast, untreated fruit displayed a slow reduction in the expression level of both genes. The expression of these genes reached the lowest levels after 6 d of shelf-life in both treatments. Remarkably, a gene encoding an isoform of expansin (*FcEXP2*) showed a similar expression profile to that of *FcPG1* and *FcPL1* (Figure 5C). The expression of a gene encoding for the enzyme xyloglucan endotransglycosylase/hydrolase 1 (*FcXTH1*), involved in molecular modifications of hemicellulose did not change its transcriptional level during the first two shelf-life days either in control or H_2_S-treated fruit (Figure 5D). From the fourth day, a strong reduction in *FcXTH1* transcript levels was observed in H_2_S treated fruit, while in contrast, in non-treated fruit, there is an increment in their levels. Furthermore, the expression of a gene that encodes for endo-β-1,4-glucanase 1 (*FcEG1*), with cellulase activity, recorded a similar pattern to that of *FcXTH1* both in H_2_S-treated and controls groups (Figure 5E); however, *FcEG1* transcripts were not detected in untreated fruit at 6 d of storage.

### 2.6. Principal Components and Correlation Analyses on Studied Parameters in H_2_S-Treated Strawberries

**Figure 6:**
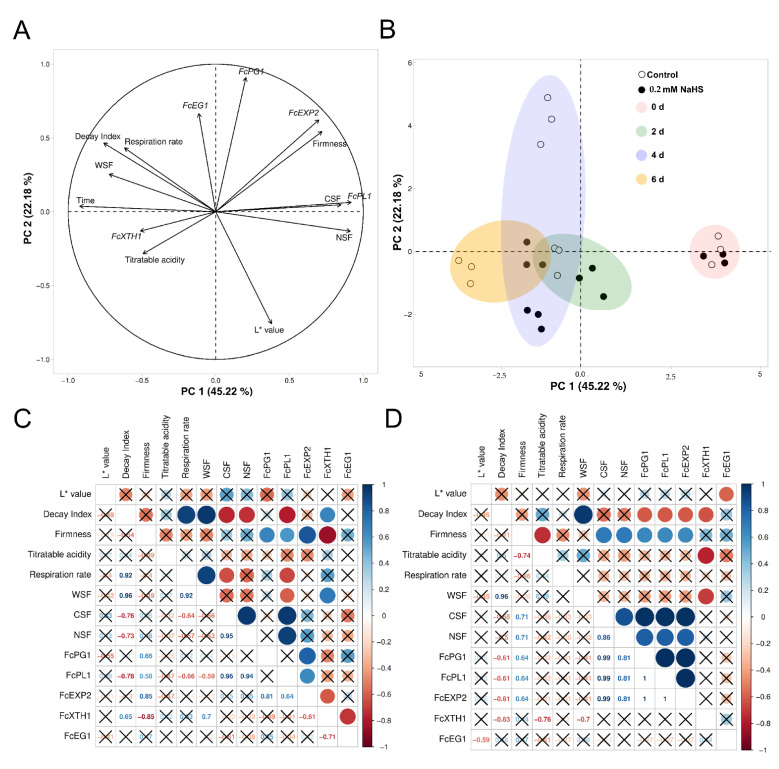
Principal component (PCA) and correlation analyses of Chilean strawberry (*Fragaria chiloensis*) fruit in response to H_2_S treatment during postharvest shelf-life: (**A**) Graph of variables; (**B**) Graph showing the response of *F. chiloensis* fruit to 0.2 mM NaHS treatment along storage time. Correlation graph of (**C**) control and (**D**) H_2_S-treated fruit. For details, see Section 4.

To understand the impact of H_2_S treatment in strawberry fruit concerning the analyzed parameters and to identify correlations between them, principal component analysis (PCA) and correlation analysis were performed. The responses are mainly explained by the first two main components (PCA 1: 45.22%; PCA 2: 22.18%) revealing that the most significant variables for the principal component 1 (PC1) were those related to CSF, NSF, and the fruit color (value of L* parameter), which showed negative correlations with treatment time, respiratory rate, and decay index. The principal component (PC2) showed a higher relationship factor for the *FcPL1*, *FcEXP2*, *FcPG1* genes, and the fruit firmness, showing a negative correlation for the *FcXTH1* gene and TA. It is worth noting the strong relationship between the ionically-bound pectin fraction (CSF) and the *FcPL1* gene. H_2_S treatments influenced the fruit responses mainly at 4 d of treatment (Figure 6B). Control fruit showed, at this time, a higher contrasting relationship for the H_2_S-treated fruit, which experienced major changes in the analyzed variables.

H_2_S-treated strawberry fruit showed a strong positive correlation amongst firmness, the CSF and covalently-bound pectin (NSF) fractions, and genes involved in pectin solubilization (*FcPG1*, *FcPL1*, *FcEXP2*), exhibiting a higher significance than the control fruit (Figure 6C,D). Furthermore, exclusively in the treated fruit, the decay index was negatively correlated to these studied variables. On the other hand, the decay index, which indicates the fruit decay, evidenced a negative correlation with the *FcXHT1* gene in the treated fruit, which showed a positive correlation in the control fruit. Additionally, this gene showed a negative correlation for the loosely-bound pectin fraction (WSF) in treated fruit. However, in the control group, the *FcXHT1* gene was positively correlated with the WSF fraction, which is also positively correlated with the respiratory rate (Figure 6C,D).

## 3. Discussion

Until now, most of the research about the role of H_2_S on the maintenance of fruit quality during postharvest storage has mainly been focused on the antioxidant system and its effect on the ethylene pathway. In this sense, the reduction in the accumulation of ROS and the increase in ascorbic acid, flavonoids, phenolics, and the enzymatic activities of ascorbate peroxidase (APX), catalase (CAT), peroxidase (POD), and superoxide dismutase (SOD) has been reported in fruits such as apple, pear, kiwifruit, grape, strawberry, and mulberry [36,37,39,40,41,42]. Regarding interference on ethylene pathway by H_2_S treatment, it has been reported in tomato, a downregulation of fruit ripening-related genes such as those encoding for ethylene-responsive transcription factors *ERF003* and *DOF22* [35]. In apple and kiwifruit, H_2_S was linked with the suppression of the expression of genes involved in ethylene biosynthesis and signal transduction [39] and the inhibition of ethylene production [41], thereby supporting the counteractive role of H_2_S in the ethylene pathway in climacteric fruits. Certainly, less is known about the specific role of H_2_S on cell wall degradation and related gene expression during postharvest of fleshy fruits. In the present work, we showed evidence about the effect of H_2_S on the pectin and hemicellulose catabolism and the expression of genes encoding for the main strawberry cell wall-modifying enzymes.

A quick decay, softening, and loss of fruit peel lightness are common processes during the senescence of *Fragaria chiloensis* fruit [43,44]. In this work, it was found that the shelf-life of Chilean strawberry fruit was extended by H_2_S treatment by an effect of optimal dose, reaching a most favorable effect with a dose of 0.2 mM NaHS donor, which was evidenced by the largest decrease in decay index and extending the shelf-life limit of treated fruit, around thrice compared to control fruit. The extended strawberry shelf-life was accompanied by a maintained firmness and color lightness, which indicates a significant delay in the senescence process of strawberry fruit tissue. These findings were consistent with previous studies [37] that have reported that H_2_S prolongs the postharvest shelf-life of strawberry (*F.* × *ananassa* ‘Bao Jiao’) by an antioxidative role in fruit. Interestingly, Hu et al. [37] recorded that the optimal dose of H_2_S donor NaHS was 0.8 mM, which is four times higher than the 0.2 mM required for the optimum effect in *F. chiloensis* fruit (Figure 1). This result might be explained by differences in the perception and signaling pathway of H_2_S of these strawberry species. It is worth noting that *F. chiloensis* is the maternal parental of *F.* × *ananassa* and the above-described differences might be derived from the genetic cross during the mating process between the parental strawberry species, i.e., *F. chiloensis* × *F. virginiana* [1]. Alternatively, the lower dosage of H_2_S required for decay control in *F. chiloensis* could also be explained by its rusticity, as it is still a native undomesticated species that preserves its natural defensive strategies [4].

The physiological events during the ripening and senescence of strawberry fruit mainly involve softening associated cell wall modification [7]. The results reported in this work have shown that H_2_S treatment delayed softening on fumigated *F. chiloensis* fruit, which remained significantly firmer than untreated fruit throughout the monitored days (Figure 3A). Similar results in firmness preservation triggered by H_2_S applications have been reported in strawberry ‘Bao Jiao’ and ‘Fengxiang’ cultivars [37,38], banana ´Brazil’ [45,46] and kiwifruit ‘Jinkui’ [41,47]. Besides, in the present research, the respiration rate and titratable acidity were also delayed by the H_2_S exposure on Chilean strawberry fruit, confirming the role of this molecule as an inhibitor of respiration rate as has been described in other non-climacteric fruits such as *F.* × *ananassa* [37,38] and mulberry (*Morus indica* ‘Dianmian-1’) [42]. It has been previously reported that the high softening rate of Chilean strawberry fruit contributes to its fast postharvest decay [12]. Furthermore, the respiration rate is an important factor in determining the postharvest deterioration of strawberry fruit [37]. Therefore, a delayed softening and respiration rate, due to the H_2_S treatment, influences an extended postharvest shelf-life by a decrease in the senescence and deterioration process.

Regarding changes in cell wall-associated polymers, the effect of H_2_S treatment on cell wall polymer solubilization during the shelf-life of Chilean strawberry fruit was investigated. Pectin solubilization of H_2_S-treated fruit was delayed compared to control fruit as loosely-bound pectin fraction (WSF) did not increase as much as in control fruit up to the fourth day, and consequently, ionically (CSF)- and covalently (NSF)-bound pectin fractions did not decrease as in control fruit (Figure 4A–D). All this evidence suggests that H_2_S fumigation delays pectin degradation in Chilean strawberry fruit during its postharvest shelf-life period, which is related to the evidenced delay in softening rate in H_2_S-treated strawberry fruit. Interestingly, differences between control and H_2_S-treated fruit were also observed in hemicellulose-related fraction (KSF) in all days of treatment (Table S1), supporting the idea that H_2_S could affect hemicellulose metabolism during postharvest storage. NaHS treatment has been recently observed altering the contents of cellulose and hemicellulose in alfalfa [48]. As far as we know, these results are the first report of the effect of H_2_S treatment on the cell wall polymer contents in fleshy fruit. Therefore, the effect of H_2_S on cell wall polymers especially hemicellulose and cellulose in fleshy fruit needs further characterization.

Plant cell wall disassembly during softening is a direct result of specific enzymatic activities, where pectin degradation plays a major role [10]. Previous reports have evidenced that decreased pectin depolymerization, by an antisense knockdown of a key pectinase gene (pectate lyase), leads to a reduction in the softening rate of strawberry fruit [13]. Thus, to gain molecular insights about how H_2_S delays the cell wall polymer solubilization, the relative expression of key genes involved in pectin and hemicelluloses degradation during strawberry postharvest shelf-life was studied. The results evidenced that H_2_S downregulates the expression of genes involved in pectin and hemicellulose metabolism. Genes encoding key enzymes involved in pectin solubilization such as polygalacturonase (*FcPG1*), pectate lyase (*FcPL1*), and an isoform of expansin (*FcEXP2*), a non-enzymatic protein associated with Chilean strawberry softening [49], exhibited a drastic and fast reduction in response to H_2_S treatment (Figure 5A–C). It has been reported that the softening rate of the Chilean strawberry fruit reflects the expression of polygalacturonase and pectate lyase genes [12]. Additionally, the expression of expansin genes in strawberry varieties is highly related to contrasting fruit firmness [11,14]. Furthermore, a higher relationship factor for the *FcPL1*, *FcEXP2*, *FcPG1* genes and the fruit firmness was observed (Figure 6A). Additionally, H_2_S-treated strawberry fruit showed a strong positive correlation between firmness, the ionically- and covalently-bound pectin fractions (CSF and NSF), and genes involved in pectin solubilization (*FcPG1*, *FcPL1*, *FcEXP2*), exhibiting a higher significance than in control fruit (Figure 6C,D). Therefore, the effects displayed by H_2_S treatment on decreasing the softening rate, pectin solubilization and the expression of genes involved in pectin depolymerization are highly associated with this previous evidence. Moreover, it has been reported that PG and PE activities are decreased in H_2_S-treated strawberry fruits, prolonging their shelf-life [37,38]. Furthermore, the expression of a gene encoding for an isoform of xyloglucan endotransglycosylase/hydrolase (*FcXTH1*), involved in molecular modifications of hemicellulose, and a gene that encodes endo-β-1,4-glucanase (*FcEG1*), with cellulase activity, did not change its transcriptional level until the fourth shelf-life day of H_2_S-treatment, exhibiting a significant increase and a strong reduction, respectively (Figure 5D,E). H_2_S treatment could probably affect the synthesis of the hemicellulosic polymers by an unknown mechanism that needs further characterization.

## 4. Materials and Methods

### 4.1. Plant Material and Treatments

Ripe *F. chiloensis* fruit were harvested from a commercial orchard at Purén, the Araucanía Region, Chile (latitude 38°04’ S; longitude 73°14’ W). The collected strawberries were immediately transported to the laboratory. Fruit of similar size and without external damage and microbial infection symptoms were selected. A total of 540 fruits were used for the following experiments.

Hydrogen sulfide (H_2_S) fumigation was performed as described by Hu et al. [37] with some modifications in sealed chambers (5 L) using sodium hydrosulfide (NaHS) as an H_2_S donor. Each chamber contained six perforated clamshells, which contained six strawberries, respectively. Initially, 200 mL of NaHS aqueous solutions at 0.2, 0.4, 0.8 and 1.2 mM were placed inside independent chambers to fumigate the fruit at 20 °C for up to 5 d. It is worth noting that the H_2_S-donor (NaHS) and control (water) solutions (200 mL) and the chamber atmosphere were renewed each 24 h, by opening the lid of treatment and control chambers. For the control treatment, water was used instead of NaHS solution. Longer treatments (up to 14 d) were performed at 0.2 mM H_2_S.

### 4.2. Evaluation of Decay Index

Decay index was determined as described by Ayala-Zavala et al. [50] and Hu et al. [37] with modifications. Fifty strawberry fruit per treatment were selected for the assessment of the decay index. Each fruit was classified into four ranks according to decay area: 0, no decay; 1, decay surface less than 10 %; 2, decay surface between 10% and 30%; 3, decay surface between 30% and 50%; 4, decay surface more than 50%. The decay examination was recorded every day using the whole set of fruit per condition. The decay index was calculated by the following equation: decay index = [∑(rank × fruit quantity per rank)/number of fruit x higher rank (4)] × 100%. The experiment was repeated in two different harvest seasons.

### 4.3. Analysis of Fruit Chromaticity

The external color of individual strawberry fruit was analyzed with a colorimeter (model CR-200, Minolta), which measure L*, a*, and b* values, where L* indicates lightness, a* indicates chromaticity on a green (−) to red (+) axis, and b* indicates chromaticity on a blue (−) to yellow (+) axis. Two measurements on each equatorial side were performed per fruit. Values reported corresponding to the mean ± SE of three fruit per experimental condition. The initial fruit L*, a*, and b* values were a mean of 61.0, 6.1, and 17.0, respectively.

### 4.4. Analysis of Fruit Respiration Rate

CO_2_ production rate (expressed in μmol CO_2_ kg^−1^ s^−1^) was determined in three independent experiments by a CO_2_ and O_2_ analyzer (BRIDGE Analyzers, Inc., Bedford Heights, OH, USA). CO_2_ percentage was measured after 1 h of incubation of three strawberry fruit in hermetic flasks. The initial fruit respiration rate had a mean of 1.0 μmol CO_2_ kg^−1^ s^−1^.

### 4.5. Fruit Firmness Measurement

Firmness (N) was measured using the FirmTech II (BioWorks, Wamego, KS, USA) provided with a flat tip of 2 cm. Samples of six strawberry fruit were evaluated per each experimental condition. Two measurements on each equatorial side were performed per fruit with a penetration depth of 1 mm. The values reported corresponds to the mean ± SE of six fruit per condition. After these analyses, the peduncle and calyx of each fruit were removed, and the fruit was cut into pieces, frozen under liquid nitrogen and stored at –80 °C for further determinations. The fruit from each experimental condition was mixed to provide a bulk of fruit samples. The initial fruit firmness had a mean of 1.7 N.

### 4.6. Determination of Soluble Solids, pH and Titratable Acidity

Two grams of frozen fruit tissue were homogenized in water with a disperser T25 digital Ultra-turrax® (IKA, Staufen, Germany) and adjusted to 25 mL final volume. The mixture was filtrated through miracloth, and the juice was analyzed for soluble solids content (SSC), pH, and titratable acidity (TA). SSC (expressed as %) was measured at 20 °C using a hand-held temperature compensated refractometer (Atago Co., Tokyo, Japan). TA (expressed as m Eq 0.1 kg^−1^ of fresh weight (FW)) was determined by titration using a pH meter and automatic titrator PH-Burette 24 (Crison, Barcelona, Spain) of an aliquot of 5 mL of strawberry juice with 20 mM NaOH until reaching pH 8.2. The pH of the juice was recorded per each replicate. Three independent fruit extractions were prepared from each biological sample, and values correspond to mean ± SE.

### 4.7. Cell Wall Extraction and Cell Wall Fractionation

Cell wall material was extracted according to Figueroa et al. [44] with some modifications. Five grams of ground frozen fruit tissue were homogenized in 40 mL of 95 % ethanol and boiled for 45 min. The insoluble material was filtered through miracloth and sequentially washed with 15 mL of boiling ethanol, 15 mL of chloroform/methanol (1:1, *v*/*v*) and 15 mL of acetone. The residue (Alcohol Insoluble Residue, AIR) was dried overnight at 37 °C and weighed. The results of three replicates per treatment were expressed as mg AIR per g^−1^ FW.

The fractionation of cell wall material was performed using a sequential chemical treatment of AIR as previously described [44]. The water-soluble, the 50 mM trans-1,2-diaminocyclohexane-N,N,N’,N’-tetraacetic acid (CDTA)-soluble, the 50 mM Na_2_CO_3_-soluble, and the 4 M KOH-soluble fractions were obtained and named as WSF, CSF, NSF, and KSF fractions, respectively. Two independent extractions were obtained from each experimental replicate. The concentration of uronic acid (UA) and neutral sugars (NS) in the different cell wall fractions were determined colorimetrically as previously described [51,52]. The results were calculated using standard curves prepared for galacturonic acid and glucose for UA and NS, respectively. Measurements were performed in triplicate, and the results were expressed as mg of galacturonic acid (UA) or glucose (NS) per g of AIR.

### 4.8. Analysis of Gene Expression

Total RNA was isolated from 2 g of frozen fruit samples using a modified CTAB method [53]. Three independent RNA extractions were prepared from each biological sample. One microgram of total RNA was treated with DNAse (Turbo DNA-free kit, Ambion®, Life Technologies) to eliminate genomic DNA. The RNA quantity and purity were estimated at 260/280 nm by NanoDrop™ 1000 Spectrophotometer (Thermo Scientific, Waltham, MA, USA). The RNA integrity was visualized in electrophoresis in 1.5 % (*w/v*) agarose gel stained with GelRedTM (Biotium, Hayward, CA, USA). The cDNA was synthesized with a first-strand cDNA synthesis kit (Thermo Scientific, Waltham, MA, USA). Quantitative reverse transcription PCR (RT-qPCR) was performed in a Stratagene Mx3000P (Agilent Technologies, Santa Clara, CA, USA). Each reaction consisted of 20 μL containing 2 μL of (1:10 diluted) cDNA, 1 μL of primer mix 10 μM, 10 μL of Sybr Green PCR Master Mix 2X (Stratagene ®, Agilent Technologies), and nuclease-free water to reach the final reaction volume. The cycling conditions were 1 cycle of denaturation at 95 °C for 5 min, followed by 40 cycles (two-segment) of amplification (95 °C/15 s, 60 °C/45 s) and a final melting cycle (95 °C/1 min, 55 °C/30 s and 95 °C/30 s). Each RT-qPCR reaction was performed in three technical replicates and the mean was used for further analysis. A control without template was included in each RT-qPCR run. Fluorescence was measured at the end of each extension step. The specific primer sequences for the genes encoding polygalacturonase 1 (*FcPG1*), pectate lyase (*FcPL*), endo-β-1,4-glucanase 1 (*FcEG1*), expansin 2 (*FcEXP2*), xyloglucan endotransglycosylase/hydrolase 1 (*FcXTH1*), and glyceraldehyde 3-phosphate dehydrogenase (*FcGAPDH*) from *F. chiloensis* were obtained from a previous report [44]. The relative expression levels were normalized by 2^−ΔΔCT^ method [54] using *GAPDH* as the reference gene. The results were expressed in arbitrary units assigning the value of one unit to time zero. The data were analyzed by Tukey test (*p* < 0.05) per time. Each value represents mean ± SD (*n* = 4).

### 4.9. Statistical Analysis

The results were compared by two-way analysis of variance (ANOVA) (time, treatment) and Duncan’s multiple range test at the 5 % level of significance. Shapiro–Wilk and Levene’s tests were used to verify normality and homoscedasticity, respectively. Principal component analysis and a Pearson correlation analysis were performed to evaluate the differences between the treatments (control and treated with H_2_S) using R 1.3.1093. The data were presented with their means and standard errors.

## 5. Conclusions

In this work, we demonstrated a novel role of H_2_S as a gasotransmitter prolonging the postharvest shelf-life of the Chilean strawberry fruit by preventing its decay and its fast softening rate through a decreased pectin degradation, which reflects the downregulation effect on the expression of key pectinase-related genes. These pieces of evidence provide useful information about the biological function of H_2_S acting as a plant gasotransmitter and transforms the Chilean strawberry fruit as an emergent model for studying the quick softening rate and decay in non-climacteric fruit. In addition, this biological system could provide, in the future, valuable information about the role of H_2_S as a cell wall modifier in plants, adding new insights into the regulation of postharvest physiology of fruit and vegetables during storage. Furthermore, the use of this elicitor emerges as a potent tool for the exogenous application of horticultural products for storage and shelf-life preservation, albeit the approval for use of H_2_S gas on fresh foods is still pending at the global level.

## Supplementary Materials

The following are available online at https://www.mdpi.com/article/10.3390/ijms221810008/s1, Table S1: Changes in total cell wall material (AIR) content and pectins and hemicellulose fractions during the shelf-life period of Chilean strawberry fruit subjected to H_2_S treatment.

## References

[1] Hancock J.F. (1999). Strawberries.

[2] Molinett S., Nuñez F., Moya-Leon M.A., Zúñiga-Hernández J. (2015). Chilean Strawberry Consumption Protects against LPS-Induced Liver Injury by Anti-Inflammatory and Antioxidant Capability in Sprague-Dawley Rats. Evid. Based Complement. Altern. Med..

[3] Mora F., Zúñiga P.E., Figueroa C.R. (2019). Genetic Variation and Trait Correlations for Fruit Weight, Firmness and Color Parameters in Wild Accessions of Fragaria chiloensis. Agronomy.

[4] Letelier L., Gaete-Eastman C., Peñailillo P., Moya-León M.A., Herrera R. (2020). Southern Species From the Biodiversity Hotspot of Central Chile: A Source of Color, Aroma, and Metabolites for Global Agriculture and Food Industry in a Scenario of Climate Change. Front. Plant Sci..

[5] Cherian S., Figueroa C.R., Nair H. (2014). ‘Movers and shakers’ in the regulation of fruit ripening: A cross-dissection of climacteric versus non-climacteric fruit. J. Exp. Bot..

[6] Fuentes L., Figueroa C.R., Valdenegro M. (2019). Recent Advances in Hormonal Regulation and Cross-Talk during Non-Climacteric Fruit Development and Ripening. Horticulturae.

[7] Moya-León M.A., Mattus-Araya E., Herrera R. (2019). Molecular Events Occurring During Softening of Strawberry Fruit. Front. Plant Sci..

[8] Figueroa N.E., Gatica-Meléndez C., Figueroa C.R. (2021). Ethylene application at the immature stage of *Fragaria chiloensis* fruit represses the anthocyanin biosynthesis with a concomitant accumulation of lignin. Food Chem..

[9] Brummell D.A., Harpster M.H. (2001). Cell wall metabolism in fruit softening and quality and its manipulation in transgenic plants. Plant Mol. Biol..

[10] Wang D., Yeats T.H., Uluisik S., Rose J.K., Seymour G.B. (2018). Fruit Softening: Revisiting the Role of Pectin. Trends Plant Sci..

[11] Dotto M., Martínez G.A., Civello P.M. (2006). Expression of expansin genes in strawberry varieties with contrasting fruit firmness. Plant Physiol. Biochem..

[12] Figueroa C.R., Pimentel P., Gaete C., Moya M., Herrera R., Caligari P.D., Moya-León M.A. (2008). Softening rate of the Chilean strawberry (Fragaria chiloensis) fruit reflects the expression of polygalacturonase and pectate lyase genes. Postharvest Biol. Technol..

[13] Santiago-Doménech N., Jimenez-Bemudez S., Matas A.J., Rose J.K.C., Blanco J.M., Mercado J.A., Quesada M.A. (2008). Antisense inhibition of a pectate lyase gene supports a role for pectin depolymerization in strawberry fruit softening. J. Exp. Bot..

[14] Ramos P., Parra-Palma C., Figueroa C.R., Zuñiga P.E., Valenzuela-Riffo F., Gonzalez J., Gaete-Eastman C., Morales-Quintana L. (2018). Cell wall-related enzymatic activities and transcriptional profiles in four strawberry (Fragaria × ananassa) cultivars during fruit development and ripening. Sci. Hortic..

[15] Rosli H., Civello P., Martínez G. (2004). Changes in cell wall composition of three Fragaria × ananassa cultivars with different softening rate during ripening. Plant Physiol. Biochem..

[16] Figueroa C.R., Rosli H., Civello P.M., Martínez G.A., Herrera R., Moya-Leon M.A. (2010). Changes in cell wall polysaccharides and cell wall degrading enzymes during ripening of *Fragaria chiloensis* and Fragaria × ananassa fruits. Sci. Hortic..

[17] Koh T.H., Melton L.D., Newman R.H. (1997). Solid-state 13C NMR characterization of cell walls of ripening strawberries. Can. J. Bot..

[18] Opazo M.C., Figueroa C.R., Henríquez J., Herrera R., Bruno C., Valenzuela P.D., Moya-Leon M.A. (2010). Characterization of two divergent cDNAs encoding xyloglucan endotransglycosylase/hydrolase (XTH) expressed in *Fragaria chiloensis* fruit. Plant Sci..

[19] Koch M.S., Erskine J.M. (2001). Sulfide as a phytotoxin to the tropical seagrass *Thalassia testudinum*: Interactions with light, salinity and temperature. J. Exp. Mar. Biol. Ecol..

[20] Wang R. (2002). Two’s company, three’s a crowd: Can H2S be the third endogenous gaseous transmitter?. FASEB J..

[21] Li L., Rose P., Moore P.K. (2011). Hydrogen Sulfide and Cell Signaling. Annu. Rev. Pharmacol. Toxicol..

[22] Aroca A., Gotor C., Bassham D.C., Romero L.C. (2020). Hydrogen Sulfide: From a Toxic Molecule to a Key Molecule of Cell Life. Antioxidants.

[23] Chen J., Wu F.-H., Wang W.-H., Zheng C.-J., Lin G.-H., Dong X.-J., He J.-X., Pei Z.-M., Zheng H.-L. (2011). Hydrogen sulphide enhances photosynthesis through promoting chloroplast biogenesis, photosynthetic enzyme expression, and thiol redox modification in *Spinacia oleracea* seedlings. J. Exp. Bot..

[24] García-Mata C., Lamattina L. (2010). Hydrogen sulphide, a novel gasotransmitter involved in guard cell signalling. New Phytol..

[25] Zhang H., Hu L.-Y., Hu K.-D., He Y.-D., Wang S.-H., Luo J.-P. (2008). Hydrogen Sulfide Promotes Wheat Seed Germination and Alleviates Oxidative Damage against Copper Stress. J. Integr. Plant Biol..

[26] Zhang P., Luo Q., Wang R., Xu J. (2017). Hydrogen sulfide toxicity inhibits primary root growth through the ROS-NO pathway. Sci. Rep..

[27] Zhang H., Hu S.-L., Zhang Z.-J., Hu L.-Y., Jiang C.-X., Wei Z.-J., Liu J., Wang H.-L., Jiang S.-T. (2011). Hydrogen sulfide acts as a regulator of flower senescence in plants. Postharvest Biol. Technol..

[28] Bloem E., Riemenschneider A., Volker J., Papenbrock J., Schmidt A., Salac I., Haneklaus S., Schnug E. (2004). Sulphur supply and infection with *Pyrenopeziza brassicae* influence L-cysteine desulphydrase activity in *Brassica napus* L.. J. Exp. Bot..

[29] Ziogas V., Molassiotis A., Fotopoulos V., Tanou G. (2018). Hydrogen Sulfide: A Potent Tool in Postharvest Fruit Biology and Possible Mechanism of Action. Front. Plant Sci..

[30] Alvarez C., Calo L., Romero L.C., Garcia I., Gotor C. (2009). An O-Acetylserine(thiol)lyase Homolog with l-Cysteine Desulfhydrase Activity Regulates Cysteine Homeostasis in Arabidopsis. Plant Physiol..

[31] Papenbrock J., Riemenschneider A., Kamp A., Schulz-Vogt H., Schmidt A. (2007). Characterization of Cysteine-Degrading and H2S-Releasing Enzymes of Higher Plants—From the Field to the Test Tube and Back. Plant Biol..

[32] Rausch T., Wachter A. (2005). Sulfur metabolism: A versatile platform for launching defence operations. Trends Plant Sci..

[33] Youssefian S., Nakamura M., Sano H. (1993). Tobacco plants transformed with the O-acetylserine (thiol) lyase gene of wheat are resistant to toxic levels of hydrogen sulphide gas. Plant J..

[34] Ge Y., Hu K.-D., Wang S.-S., Hu L.-Y., Chen X.-Y., Li Y.-H., Yang Y., Yang F., Zhang H. (2017). Hydrogen sulfide alleviates postharvest ripening and senescence of banana by antagonizing the effect of ethylene. PLoS ONE.

[35] Yao G.-F., Li C., Sun K.-K., Tang J., Huang Z.-Q., Yang F., Huang G.-G., Hu L.-Y., Jin P., Hu K.-D. (2020). Hydrogen Sulfide Maintained the Good Appearance and Nutrition in Post-harvest Tomato Fruits by Antagonizing the Effect of Ethylene. Front. Plant Sci..

[36] Ni Z.-J., Hu K.-D., Song C.-B., Ma R.-H., Li Z.-R., Zheng J.-L., Fu L.-H., Wei Z.-J., Zhang H. (2016). Hydrogen Sulfide Alleviates Postharvest Senescence of Grape by Modulating the Antioxidant Defenses. Oxidative Med. Cell. Longev..

[37] Hu L.-Y., Hu S.-L., Wu J., Li Y.-H., Zheng J.-L., Wei Z.-J., Liu J., Wang H.-L., Liu Y.-S., Zhang H. (2012). Hydrogen Sulfide Prolongs Postharvest Shelf Life of Strawberry and Plays an Antioxidative Role in Fruits. J. Agric. Food Chem..

[38] Zhang C., Shi J.Y., Zhu L.P., Li C.L., Wang Q.G. (2014). Cooperative effects of hydrogen sulfide and nitric oxide on delaying sof-tening and decay of strawberry. Int. J. Agric. Biol. Eng..

[39] Zheng J.L., Hu L.Y., Hu K.D., Wu J., Yang F., Zhang H. (2016). Hydrogen sulfide alleviates senescence of fresh-cut apple by reg-ulating antioxidant defense system and senescence-related gene expression. Hortscience.

[40] Hu K.-D., Wang Q., Hu L.-Y., Gao S.-P., Wu J., Li Y.-H., Zheng J.-L., Han Y., Liu Y.-S., Zhang H. (2014). Hydrogen Sulfide Prolongs Postharvest Storage of Fresh-Cut Pears (*Pyrus pyrifolia*) by Alleviation of Oxidative Damage and Inhibition of Fungal Growth. PLoS ONE.

[41] Zhu L., Wang W., Shi J., Zhang W., Shen Y., Du H., Wu S. (2014). Hydrogen sulfide extends the postharvest life and enhances antioxidant activity of kiwifruit during storage. J. Sci. Food Agric..

[42] Hu H., Shen W., Li P. (2013). Effects of hydrogen sulphide on quality and antioxidant capacity of mulberry fruit. Int. J. Food Sci. Technol..

[43] Saavedra G.M., Figueroa N.E., Poblete L.A., Cherian S., Figueroa C.R. (2016). Effects of preharvest applications of methyl jasmonate and chitosan on postharvest decay, quality and chemical attributes of *Fragaria chiloensis* fruit. Food Chem..

[44] Figueroa C.R., Opazo M.C., Vera P., Arriagada O., Díaz M., Moya-León M.A. (2012). Effect of postharvest treatment of calcium and auxin on cell wall composition and expression of cell wall-modifying genes in the Chilean strawberry (*Fragaria chiloensis*) fruit. Food Chem..

[45] Luo Z., Li D., Du R., Mou W. (2015). Hydrogen sulfide alleviates chilling injury of banana fruit by enhanced antioxidant system and proline content. Sci. Hortic..

[46] Li D., Limwachiranon J., Li L., Du R., Luo Z. (2016). Involvement of energy metabolism to chilling tolerance induced by hydrogen sulfide in cold-stored banana fruit. Food Chem..

[47] Yonggen S., Wei W., Weil Z., Liqin Z. (2015). Hydrogen sulfide facilitating enhancement of antioxidant ability and maintenance of fruit quality of kiwifruits during low temperature storage. Trans. Chin. Soc. Agric. Eng..

[48] Li J., Wang X., Wang X., Ma P., Yin W., Wang Y., Chen Y., Chen S., Jia H. (2020). Hydrogen sulfide promotes hypocotyl elongation via increasing cellulose content and changing the arrangement of cellulose fibrils in alfalfa. J. Exp. Bot..

[49] Figueroa C.R., Pimentel P., Dotto M.C., Civello P.M., Martínez G.A., Herrera R., Moya-León M.A. (2009). Expression of five expansin genes during softening of *Fragaria chiloensis* fruit: Effect of auxin treatment. Postharvest Biol. Technol..

[50] Zavala J.F.A., Wang S.Y., Wang C.Y., González-Aguilar G.A. (2004). Effect of storage temperatures on antioxidant capacity and aroma compounds in strawberry fruit. LWT.

[51] Blumenkrantz N., Asboe-Hansen G. (1973). New method for quantitative determination of uronic acids. Anal. Biochem..

[52] Yemm E.W., Willis A.J. (1954). The estimation of carbohydrates in plant extracts by anthrone. Biochem. J..

[53] Gasic K., Hernandez A., Korban S.S. (2004). RNA extraction from different apple tissues rich in polyphenols and polysaccharides for cDNA library construction. Plant Mol. Biol. Rep..

[54] Livak K.J., Schmittgen T.D. (2001). Analysis of Relative Gene Expression Data Using Real-Time Quantitative PCR and the 2−ΔΔCT Method. Methods.

