# Supplementary material for: Postharvest Treatment of Hydrogen Sulfide Delays the Softening of Chilean Strawberry Fruit by Downregulating the Expression of Key Genes Involved in Pectin Catabolism
Source: Int J Mol Sci. 2021 Sep 16;22(18):10008. doi: 10.3390/ijms221810008 (PMC8469075; doi:10.3390/ijms221810008)
Supplement: Supplementary file 1 [file ijms-22-10008-s001.zip › ijms-1342873-supplementary.pdf]

## Supplementary Material

**Table S1.** Changes in total cell wall material (AIR) content and pectins and hemicellulose fractions during the shelf-life period of Chilean strawberry fruit subjected to $H_2S$ treatment. The content of uronic acids (UA) of water-soluble (WSF), CDTA-soluble (CSF) and $Na_2CO_3$-soluble (NSF) pectin fractions and neutral sugars (NS) of KOH-soluble (KSF) fraction (hemicellulose) are indicated.

| **Day** | **Treatment** | **Cell Wall Material (AIR) (mg g$^{-1}$ FW)** | **Cell Wall Fractions (mg g$^{-1}$ AIR)** | | | |
|---|---|---|---|---|---|---|
| | | | WSF (UA) | CSF (UA) | NSF (UA) | KSF (NS) |
| 0 | Non-treated | 13.90 ± 1.53 | 20.00 ± 0.88 | 6.38 ± 0.33 | 13.37 ± 2.42 | 10.09 ± 2.42 |
| 2 | $H_2S$ | 18.79 ± 1.17 a | 18.21 ± 20.03 a | 2.01 ± 0.11 a | 9.10 ± 2.01 a | 11.03 ± 0.50 b |
| | Control | 18.50 ± 3.79 a | 21.26 ± 1.17 a | 2.67 ± 0.39 a | 6.27 ± 0.74 a | 22.17 ± 6.85 a |
| 4 | $H_2S$ | 17.24 ± 1.77 b | 26.28 ± 2.24 b | 1.61 ± 0.07 b | 7.49 ± 0.96 a | 14.79 ± 1.98 b |
| | Control | 26.95 ± 4.46 a | 32.78 ± 1.00 a | 2.42 ± 0.20 a | 6.67 ± 0.76 a | 32.23 ± 4.30 a |
| 6 | $H_2S$ | 13.21 ± 2.03 b | 39.75 ± 1.24 a | 2.39 ± 0.18 a | 8.92 ± 0.84 a | 19.26 ± 2.45 b |
| | Control | 24.72 ± 1.51 a | 36.44 ± 2.24 b | 3.01 ± 0.36 a | 6.47 ± 0.43 b | 41.91 ± 3.27 a |

Data indicate the mean of three replicates ± S.D. Different letters indicate significant differences between treatments at each day ($p \leq 0.05$). FW, fresh weight.
